# The N-terminal domain of Type IV-A1 CRISPR-associated DinG is vulnerable to proteolysis

**DOI:** 10.17912/micropub.biology.001226

**Published:** 2024-06-05

**Authors:** Thomson Hallmark, Andrew A. Williams, Olivine Redman, Brendon Guinn, Calvin Judd, Ryan N. Jackson

**Affiliations:** 1 Department of Chemistry and Biochemistry, Utah State University, Logan, Utah, United States

## Abstract

CasDinG is an ATP-dependent 5′-3′ DNA helicase essential for bacterial Type IV-A1 CRISPR associated immunity. CasDinG contains an essential N-terminal domain predicted to bind DNA. To better understand the role of the N-terminal domain, we attempted to co-crystallize CasDinG with DNA substrates. We successfully crystallized CasDinG in a tightly packed, crystal conformation with previously unobserved unit cell dimensions. However, the structure lacked electron density for a bound DNA substrate and the CasDinG N-terminal domain. Additionally, the tight crystal packing disallowed space for the N-terminal domain, indicating that the N-terminal domain was proteolyzed before crystallization. Follow up experiments revealed that the N-terminal domain of CasDinG is proteolyzed after a few days at room temperature, but is protected from proteolysis at 4°C. These data provide a distinct x-ray crystal structure of CasDinG and indicate the essential N-terminal domain of CasDinG is prone to proteolysis.

**Figure 1. Characterization of CasDinG crystals.**
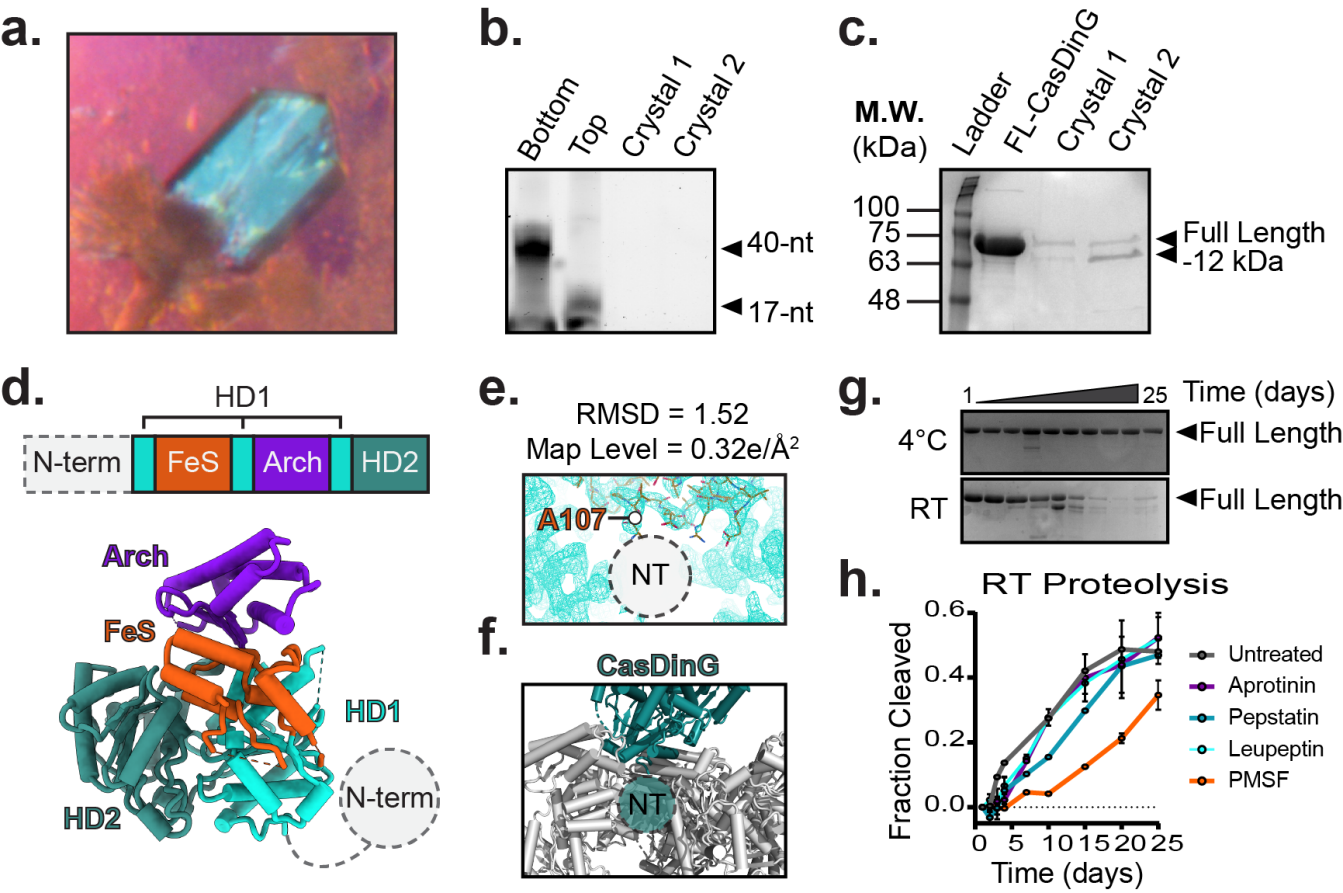
**a.** ) Photograph of CasDinG crystal. **b.** ) Analysis of DNA content of crystals by urea-PAGE. **c.** ) Analysis of protein content of crystals by SDS-PAGE. **d.** ) Cartoon model *Pa* CasDinG atomic coordinates with primary sequence (above) and model colored by domain. **e.** ) Electron density of *Pa* CasDinG with the most N-terminal resolved residue (A107) indicated. No density is observed that corresponds to the N-terminal domain. **f.** ) CasDinG (teal) with symmetry mates (grey). No space is available for the N-terminal domain in the crystal packing. **g.** ) *Pa* CasDinG proteolysis time-course at 4℃ (top) and room temperature (bottom). **h.** ) Time course of *Pa* CasDinG room temperature proteolysis in the presence of different protease inhibitors.

## Description

CRISPR - Cas RNA-guided adaptive immune systems defend bacteria against plasmids and viruses (Hille et al., 2018) . Of the six defined types of CRISPR systems, type IV systems are the least characterized (Makarova et al., 2020; Pinilla-Redondo et al., 2020; Taylor et al., 2021) . To provide immunity against plasmids, it is proposed that type IV-A systems bind DNA with an RNA-guided multi-protein Csf complex, and then recruit an accessory helicase called CasDinG to the DNA (Crowley et al., 2019; Özcan et al., 2019; Zhou et al., 2021; Guo et al., 2022). It is supposed that, once loaded onto Csf-complex-bound DNA, CasDinG uses its ATP-dependent 5′-3′ helicase activity to silence transcription of nearby genes, mediating immunity (Guo et al., 2022; Cui et al., 2023; Domgaard et al., 2023) . Multi-subunit Type I CRISPR systems execute immunity in a similar fashion, targeting dsDNA with a multi-subunit RNA-guided complex, and recruiting a helicase (Cas3) to the bound DNA. However, unlike CasDinG, Cas3 unwinds DNA with the opposite polarity (3’-5’) and contains an N-terminal HD nuclease domain that cleaves the DNA target (Huo et al., 2014; Redding et al., 2015) . Some CasDinG variants have been shown to cleave dsDNA similar to Cas3 with a C-terminal HNH nuclease domain (Altae-Tran et al., 2023) . However, *in vivo* studies indicate that many CasDinG enzymes do not degrade the DNA target, but instead knock-down RNA transcription (Guo et al., 2022) .

The CasDinG protein from Type IV-A1 systems (eg. *Pseudomonas aeruginosa* , CasDinG ( *Pa* CasDinG)) has an N-terminal domain of unknown function that is essential for Type IV-A1 immunity (Guo et al., 2022; Domgaard et al., 2023) . Crystal structures of *Pa* CasDinG have been solved, but none resolved the N-terminal domain (Cui et al., 2023; Domgaard et al., 2023) . AlphaFold2 predictions suggested that the N-terminal domain forms a knot-fold, characteristic of DNA binding proteins, suggesting the domain may bind dsDNA and extend the range of transcription knockdown (Domgaard et al., 2023) . However, without an experimentally determined structure, the function for this essential domain remains unknown. In an attempt to determine the structure of the N-terminal domain we solved the structure of a new crystal form of *Pa* CasDinG where overhang dsDNA substrates were included in our crystal conditions.

Our previous AlphaFold2 analysis suggested the N-terminal domain might bind to dsDNA. Thus, we hypothesized that adding dsDNA to a crystallization condition might stabilize the previously flexible N-terminal domain. To test our hypothesis, we screened crystals of *Pa* CasDinG grown in the presence of a 5’-overhang duplex DNA substrate. Orthorhombic crystals grew from precipitate after two weeks ( **Figure 1a** ). The morphology of this crystal condition was different from previously reported Apo *Pa* CasDinG crystals, suggesting that DNA could be bound or that the N-terminal domain could be resolved ( **Table 1** ). To verify that both DNA strands of the overhang substrate were included in the crystal, we looped and washed several crystals then probed for the presence of DNA with denaturing urea-PAGE ( **Figure 1b** ). We found no evidence of DNA substrate in any of the crystals screened. To probe the protein component of the crystals we analyzed them by SDS-PAGE ( **Figure 1c** ). We found that *Pa* CasDinG in the crystals had been proteolyzed corresponding to a ~12 kDa shift in molecular weight.

**Table:** 

**Table 1: Comparison of Crystal Geometries**			
**PDBid**	**Conditions**	**Spacegroup**	**Ref** .
**8V44**	0.1 M Tris-HCl pH 8.0, 22.5% PEG3350	P 21 21 21	this work
**8E2W**	225 mM Imidazole pH 8.0, 3.5% PEG8000	P 65	Domgaard, 2023
**7XEX**	0.1 M HEPES pH 7.4, 8% PEG6000	C 1 2 1	Cui, 2023
**7XF0**	0.1 M HEPES pH 7.4, 8% PEG6000	I 1 2 1	Cui, 2023

To understand how the observed proteolysis might impact the structure of *Pa* CasDinG, we screened crystals for diffraction. *Pa* CasDinG crystals frozen in saturated sucrose cryo-protectant diffracted to ~3.0 Å, while no diffraction was observed for crystals cryo-protected in 25% glycerol. The diffraction pattern corresponded to a unit cell of 67.04 Å x 84.01 Å x 97.619 Å and a P 21 21 21 space group, distinct from the unit cells of previously solved crystal structures ( **Table 1** , **Table 2** ). Matthews probability coefficient suggested a single solution of 1 CasDinG subunit in the unit cell with a solvent content of 29.75% (Weichenberger & Rupp, 2014) . This was in the bottom quartile of solvent content for this resolution range, suggesting that CasDinG was tightly packed in this crystal morphology.

**Table:** 

**Table 2. Data collection and refinement statistics**		
Dataset		CasDinG
Beamline		USU X-ray source
Space group		p 21 21 21
Cell dimensions:		
	a, b, c (Å)	67.04, 84.01, 97.619
	α, β, γ ( ˚ )	90, 90, 90
Wavelength (Å)		1.5
Resolution (Å)		28.76 - 2.902 (3.005 - 2.902)
Wilson B-factor		58.16
Observations		926399
Unique reflections		10,621 (521)
Redundancy		2.8 (1.5)
Completeness (%)		83.39 (42.60)
No. reflections		10592 (521)
Refinement R _work_ /R _free_		0.2254/0.2771 (0.3240/0.3724)
No. atoms:		
	Protein	4317
	Water	0
	Ligands	0
B-factor		
	mean	47.67
R.M.S. deviations:		
	Bond length (Å)	0.005
	Bond angles ( ˚ )	0.78
Ramachandran (%):		
	Favored	97.4
	Allowed	2.6
	Outliers	0
Clashscore		8.27

Phases were solved by molecular replacement using Apo *Pa* CasDinG (PDBid: 8E2W) as a starting model and the program Phaser (McCoy et al., 2007) . Consistent with the Mathews coefficient, the solution revealed tight crystal packing with narrow solvent channels. The structure of *Pa* CasDinG revealed two helicase domains (HD1 and HD2) and two accessory domains (FeS-like and Arch domains), that aligned well (RMSD = 0.547 Å) with other models of Apo *Pa* CasDinG (Cui et al., 2023; Domgaard et al., 2023) ( **Figure 1d** ). Consistent with our Urea-PAGE analysis, no density for nucleic acid was observed. Additionally, no density was observed for the N-terminal domain of *Pa* CasDinG, despite the tight crystal packing, which we had hoped would constrain the previously observed flexibility of the domain. ( **Figure 1e** ). Indeed, the *Pa* CasDinG subunits were not oriented in the crystal lattice to accommodate the size of the N-terminal domain ( **Figure 1f** ). We observed that proteolysis of the N-terminal domain (~11.3 kDa) is consistent with the shift of the *Pa* CasDinG band seen on SDS-PAGE when crystals were compared to full length *Pa* CasDinG, suggesting that proteolysis of the N-terminal domain is required for crystallization in this condition.

Because the N-terminal domain is essential for Type IV-A1 system activity, we wanted to know how quickly the N-terminal domain is proteolyzed from recombinant *Pa* CasDinG. To test the rate of proteolysis, we freshly purified *Pa* CasDinG and measured proteolysis over 25 days at either 4°C or room temperature (RT) ( **Figure 1g** ). We found that *Pa* CasDinG stored at 4°C remained unproteolyzed even after 25 days while proteins at RT were significantly proteolyzed after just 7 days. We wondered if including protease inhibitors might prolong the life of full length *Pa* CasDinG. We tested the ability of aprotinin, pepstatin, leupeptin and phenylmethylsulfonyl fluoride (PMSF) to inhibit proteolysis of the N-terminal domain and found that only the inclusion of PMSF significantly prolonged the life-time of full length *Pa* CasDinG at room temperature ( **Figure 1h** ).

These data reveal that the N-terminal domain of CasDinG is vulnerable to proteolysis at temperatures above 4°C. Because the N-terminal domain is essential for the *in vivo* function of the type IV-A1 CRISPR system in *Pseudomonas aeruginosa* , considering the potential proteolysis of the N-terminal domain will be critical for *in vitro* characterization (Crowley et al., 2019; Domgaard et al., 2023) . Furthermore, future structural studies aimed at characterizing the N-terminal domain of CasDinG should consider the effect of proteolysis when screening crystal conditions. While the function of the N-terminal domain of CasDinG remains a mystery, we provide necessary and useful insight into factors that may impact future N-terminal domain characterization.

## Methods

*Expression and Purification of CasDinG*

CasDinG was expressed on Strep II TEV ligation independent cloning (LIC) vector (2R-T). This vector was transformed into *E. coli* BL21 HMS174(DE3) chemically competent cells (Novagen). A colony was picked and placed into an overnight outgrowth in Luria-Bertani (LB) media (Fisher) at 37°C and 200 rpm. Growth media was prepared in a 2.8L flask with 1 L of LB medium supplemented with 1 ml of 1000× metals mix (0.1 M FeCl _3_ –6H _2_ O, 1 M CaCl _2_ , 1 M MnCl _2_ -4H _2_ O, 1 M ZnSO _4_ –7H _2_ O, 0.2 M CoCl _2_ –6H _2_ O, 0.1 M CuCl _2_ –2H _2_ O, 0.2 M NiCl _2_ –6H _2_ O, 0.1 M Na _2_ MoO _4_ –2H _2_ O, 0.1 M Na _2_ SeO _3_ –5H _2_ O, 0.1 M H _3_ BO _3_ ) and 1 ml of 1000× ampicillin (100mg/mL). This flask was inoculated with 20 ml of overnight starter. Cells were grown to an optical density between 1.0 and 1.3 OD _600_ at 37°C and 200 rpm, then induced with a final concentration of 0.5 mM IPTG (isopropyl β-D-1-thiogalactopyranoside), while dropping the temperature to 18°C and maintaining 200 rpm. After 5 h, cells were harvested via high-speed centrifugation and stored at –80°C.

Pelleted cells were resuspended in 50mL lysis/wash buffer (100mM Tris pH 8.0, 500mM NaCl, 1mM TCEP) supplemented with 0.5µg/mL aprotinin, 0.5µg/mL leupeptin, 0.7µg/mL pepstatin A, and 1mM AEBSF. Cells were lysed by sonication at a 5/50 duty cycle for approximately 10 minutes in 30 seconds on, 30 seconds rest intervals. Lysate was clarified by centrifugation at 16000 rpm for 35 minutes. The clarified lysate was then combined with 2-3mL of Streptactin XT 4-flow resin (IBA) in a column and allowed to batch bind for 45 minutes.

The resin was then washed with 300mL wash buffer (100mM Tris, pH 8.0, 500mM NaCl, 1mM TCEP). CasDinG was eluted from the resin by first adding 2mL of elution buffer (100mM Tris, pH 8.0, 150mM NaCl, 50mM biotin, 1mM TCEP) and eluting by gravity to equilibrate the column. Elution continued by adding 5mL of elution buffer to the column, swirling it with a pipet tip, and allowing it to incubate for 10 minutes before eluting by gravity. This step was repeated four more times for a total of five 5mL elutions (Extended Data Figure 1A ).

Elutions containing CasDinG were pooled together and desalted over a HiPrep 26/10 Desalting column (Cytiva) into low salt buffer (100mM Tris, pH 8.0, 20mM NaCl, 1mM TCEP). Resulting desalted CasDinG was subsequently loaded onto a HiTrap 5mL Heparin HP column (Cytiva) and washed with 5 column volumes of low salt buffer. CasDinG was eluted from this column using a step gradient to 50% high salt buffer (100mM Tris, pH 8.0, 1M NaCl, 1mM TCEP) (Extended Data Figure 1B ). Protein was then concentrated to a volume of 1mL and purified overnight by size exclusion chromatography using a HiLoad 26/60 Superdex 200 Prep Grade column (Cytiva) (Extended Data Figure 1C -D).

*Overhang DNA substrate annealing*

DNA duplex was made by adding equal volumes of 1mM stocks along with 1/10 volume of NEB buffer 2.1.A SimpliAmp thermal cycler (ThermoScientific) was used to heat oligos to 95℃ for 10 minutes followed by slowly cooling to 4.0℃ by 1.0℃/min.

*Crystallization and Structure Determination*

CasDinG:DNA complexes were prepared by concentrating Strep-tagged CasDinG to 10 mg/mL then adding 1 ⁄ 3 volume annealed overhang DNA substrate for a final concentration of ~7.5 mg/mL CasDinG and 112 µM DNA. CasDinG and DNA substrates were incubated together for 20 minutes at room temperature before setting up crystal trays. Crystals were grown with hanging drop vapor diffusion at room temperature. The crystal used for structure determination was retrieved from a drop set-up with 1 uL of protein solution to 1.5 uL mother liquor (0.1 M Tris base (ThermoFischer) pH 8.0 and 22.5% PEG3350 (SigmaAldrich)). Crystals were cryoprotected with saturated sucrose dissolved in mother liquor, mounted in a loop and cooled to 100 K in liquid nitrogen. Diffraction data were collected at 85K using a Micromax-007 HF rotating anode X-ray generator (Rigaku, Tokyo Japan) set to a wavelength of 1.0 Å, and detected using an Raxis IV++ image plate (Rigaku, Tokyo Japan). The data were indexed, integrated and scaled in HKL-3000 to 2.9 A resolution with the space group P 21 21 21 (Minor et al., 2006) . Phases were determined by molecular replacement in Phaser with a previously solved model of CasDinG (PDB: 8E2W) (Bunkóczi et al., 2013; Domgaard et al., 2023; McCoy et al., 2007). Model building was performed in Coot (Emsley et al., 2010) , structures were refined using PHENIX and validation was performed using Molprobity within PHENIX and PDB deposition servers (Liebschner et al., 2019) .

*Screening Crystals for bound DNA*

Crystals grown from drops containing CasDinG and annealed, overhang DNA substrate made up of 40-nt oligo (bottom strand) and 17-nt oligo (top strand) (See Reagents table). Relatively large crystals grew after ~2 weeks and were dissolved in 30uL of nuclease free water. DNA was then extracted from the crystal by phenol:chloroform extraction, combined with equal volume of 50% glycerol and run on 12% denaturing urea-PAGE. Nucleic acids were visualized by SybrGold () staining using a ChemiDoc MP Imaging System (Bio-Rad, Hercules CA, USA).

*SDS-PAGE analysis of CasDinG Crystals*

Crystals were grown as described above and washed in mother liquor before being dissolved in 30uL of nuclease free water. Dissolved crystals were then combined with SDS loading buffer (50mM Tris-HCl, 100mM dithiothreitol, 2.0% sodium dodecyl sulfate, 1.5mM bromophenol blue, 1.1 M glycerol). Protein in crystals was compared to full-length CasDinG purification samples on 12% acrylamide SDS-PAGE. Proteins were visualized by zinc staining as described by (Fernandez-Patron et al., 1992) and imaged with a ChemiDoc MP Imaging System (Bio-Rad, Hercules CA, USA).

*Proteolysis time course assays* .

To investigate CasDinG proteolysis over time, 1050uL samples of freshly purified CasDinG at a concentration of 0.4mg/mL were taken. Two sets of five samples were taken, and four of these samples were supplemented with one of four protease inhibitors: aprotinin (0.5µg/mL), pepstatin A (0.7µg/mL), leupeptin (0.5µg/mL), or PMSF (1mM). One set of five samples were kept at room temperature and the other set was kept at 4℃. At each designated time point post-SEC purification (1, 2, 3, 4, 5, 7, 10, 15, 20, and 25 days), three 30uL samples were taken from each tube of CasDinG with or without protease inhibitor and added to 10uL SDS loading buffer and heated to 95℃ for 5 minutes. After the complete 25 day time course, samples were run on an SDS PAGE gel and imaged. Bio-Rad Image Lab software was used to conduct densitometry analyses on the gels (Image Lab Software (RRID:SCR_014210)). Percent cleaved time course graphs were derived from these analyses and plotted in GraphPad Prism.

*Protein structure visualization.*

Atomic coordinates of *Pa* CasDinG are modeled in ChimeraX ( **Figure 1d** ) (Goddard et al., 2018; Meng et al., 2023; Pettersen et al., 2021) , Coot ( **Figure 1e** ) (Emsley et al., 2010; Emsley & Cowtan, 2004) and Pymol ( **Figure 1f** ). Graphs of proteolysis data were made in GraphPad Prism. Figures were compiled in Adobe Illustrator.

## Reagents

**Table:** 

Reagent	Source	
Tris Base	Fisher Bioreagents (Pittsburgh PA, USA)	
Sodium Chloride	Fisher Chemical (Pittsburgh PA, USA)	
Glycerol	Pharmco (Brookfield CT, USA)	
Biotin	IBA Lifesciences (Göttingen Germany)	
Tris(2-carboxyethyl)phospine hydrochloride (TCEP-HCl)	GoldBio (St. Louis MO, USA)	
Luria-Bertani (LB) Media	Fisher Scientific (Waltham MA, USA)	
Ampicillin	GoldBio (St. Louis MO, USA)	
Isopropyl β- d-1-thiogalactopyranoside (IPTG)	Fisher Scientific (Waltham MA, USA)	
Iron (III) Chloride Hexahydrate	Strem Chemicals Inc. (Newburyport MA, USA)	
Calcium Chloride	Acros Organics (Geel, Belgium)	
Manganese (II) Chloride Tetrahydrate	Fisher Chemical (Pittsburgh PA, USA)	
Zinc Sulfate Heptahydrate	Fisher Scientific (Waltham MA, USA)	
Cobalt (II) Chloride Hexahydrate	TCI (Tokyo, Japan)	
Nickel (II) Chloride Hexahydrate	Chem-Impex Intl Inc. (Wood Dale IL, USA)	
Sodium Molybdate Dihydrate	Fisher Chemical (Pittsburgh PA, USA)	
Sodium Selenate Pentahydrate	Honeywell Research Chemicals (Morris Plains NJ, USA)	
Boric Acid	Sigma-Aldrich (Darmstadt, Germany)	
Aprotinin	GoldBio (St. Louis MO, USA)	
Leupeptin	Alfa Aesae (Ward Hill MA, USA)	
Pepstatin A	Fisher Scientific (Waltham MA, USA)	
AEBSF	GoldBio (St. Louis MO, USA)	
Phenylmethylsulfonyl fluoride	GoldBio (St. Louis MO, USA)	
PEG3350	Sigma-Aldrich (Darmstadt, Germany)	
Sucrose	Avantor Materials (Radnor PA, USA)	
Dithiothreitol (DTT)	GoldBio (St. Louis MO, USA)	
Sodium Dodecyl Sulfate (SDS)	Fisher Bioreagents (Pittsburgh PA, USA)	
Bromophenol Blue	GoldBio (St. Louis MO, USA)	
40% Acrylamide:Bisacrylamide Solution (19:1)	Fisher Bioreagents (Pittsburgh PA, USA)	
N, N, N’, N’-tetramethylethylenediamine (TEMED)	Fisher Bioreagents (Pittsburgh PA, USA)	
Ammonium Persulfate (APS)	Sigma-Aldrich (Darmstadt, Germany)	
Urea	Fisher Chemical (Pittsburgh PA, USA)	
Ethylenediaminetetraacetic acid (EDTA)	Fisher Bioreagents (Pittsburgh PA, USA)	
Imidazole	Sigma-Aldrich (Darmstadt, Germany)	
Sybr Gold	Invitrogen (Carlsbad CA, USA)	
NEB Buffer 2.1	New England Biolabs (Ipswich MA, USA)	
		
Cell Strain	Genotype	Source
*E. coli* BL21 HMS174 (DE3)	F ^-^ *recA1 hsdR* (r _K12_^-^ m _K12_^+^ ) (Rif ^R^ )	Novagen
		
Plasmids	Genotype	Description
2R-T PaCasDinG	pET-StrepII-dinG	pET N-terminal Strep II tag *Pseudomonas aeruginosa* CasDinG expression plasmid.
		
Oligonucleotides	Sequence	Source
Top Strand	5’-TCGTCACCAGTACAAAC-3’	Integrated DNA Technologies (IDT)
Bottom Strand	5’-TTTTTTTTTTTTTTTTGTTTGTACTGGTGACGA-3’	Integrated DNA Technologies (IDT)
